# Targeted combination chemotherapy effective in nuclear protein in testis carcinoma of lung origin: A case report and review of the literature

**DOI:** 10.1097/MD.0000000000038881

**Published:** 2024-12-27

**Authors:** Ran Li, Ye Zhang, Qian Liu, Aiqin Gao, Qi Dang

**Affiliations:** a Shandong Second Medical University, Weifang, China; b Department of Thoracic Radiation Oncology, Shandong Cancer Hospital and Institute, Shandong Academy of Medical Sciences, Shandong First Medical University, Jinan, China; c Department of Oncology, Phase I Clinical Trial Center, Shandong Cancer Hospital and Institute, Shandong First Medical University and Shandong Academy of Medical Sciences, Jinan, China.

**Keywords:** midline carcinoma, nuclear protein of testis carcinoma, NUT carcinoma, pulmonary, targeted therapy

## Abstract

**Rationale:**

Nuclear protein in testis carcinoma (NC) is a rare, aggressive, poorly differentiated squamous cell carcinoma. By reviewing the entire treatment process of the patient, we aim to explore the treatment experience of NC.

**Patient concerns:**

We report the case of a 27-year-old female patient with NC whose initial symptoms were occasional cough and chest tightness with abdominal distension for more than half a month without any other specific discomfort.

**Diagnoses:**

Computed tomography showed right lung hilar and right middle and lower lobe mass, malignant, right hilar and mediastinal lymph node metastasis, and bilateral cystic solid masses in the adnexal region, malignant possibility. Pathological diagnosis showed nuclear protein in the testis (+).

**Interventions:**

After the failure of first-line chemotherapy with immunocombination, second-line chemotherapy was switched to bevacizumab, which resulted in a progression-free survival of 6 months.

**Outcomes:**

The disease then reprogressed, and she died on November 7, 2022.

**Lessons:**

The patient achieved survival of nearly 1 year on multiple courses of therapy, well beyond the currently reported median survival. The patient achieved a 6-month progression-free survival, suggesting that combination therapy with antivascular endothelial growth factor class-targeted agents is a potential approach.

## 1. Introduction

Nuclear protein in testis (NUT) carcinoma is a rare, aggressive, poorly differentiated squamous cell carcinoma.^[1]^ Since the initial identification of the first t(15;19)(q15;p13) chromosomal translocation in thymic carcinoma in 1991, NUT carcinoma has been assumed to be a tumor inextricably linked to midline structures, hence the term “NUT midline carcinoma.”^[2]^ NUT carcinoma (NC) typically develops in the sinus region^[3]^ but can also develop in the lung and pelvis.^[4]^ NC has a dismal prognosis, with a median overall survival (OS) and event-free survival of 6.5 and 4.6 (95% confidence interval, 3.8–6.2).^[5]^

## 2. Medical record introduction

**Figure 1.**
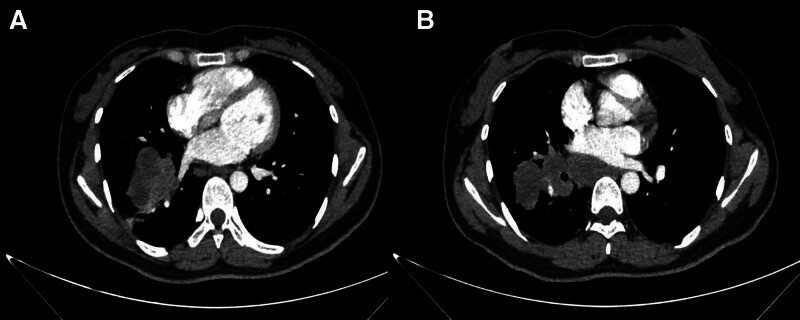
November 28, 2021. Lung computed tomography: masses in the right hilar and lower middle lobe of the right lung, considered malignant. Right hilar and mediastinal lymph node metastasis. (A) Primary tumor. (B) Maximum cross-section of tumor.

**Figure 2.**
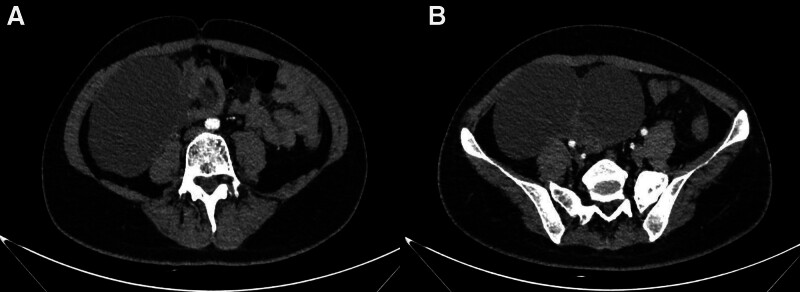
November 28, 2021. Pelvis computed tomography: bilateral cystic solid masses in the adnexal region, considered to be of high malignant potential, with possible cystic adenocarcinoma. Pelvic and abdominal fluid accumulation. (A) Pelvic metastasis. (B) Maximum cross-section of pelvic metastases.

**Figure 3.**
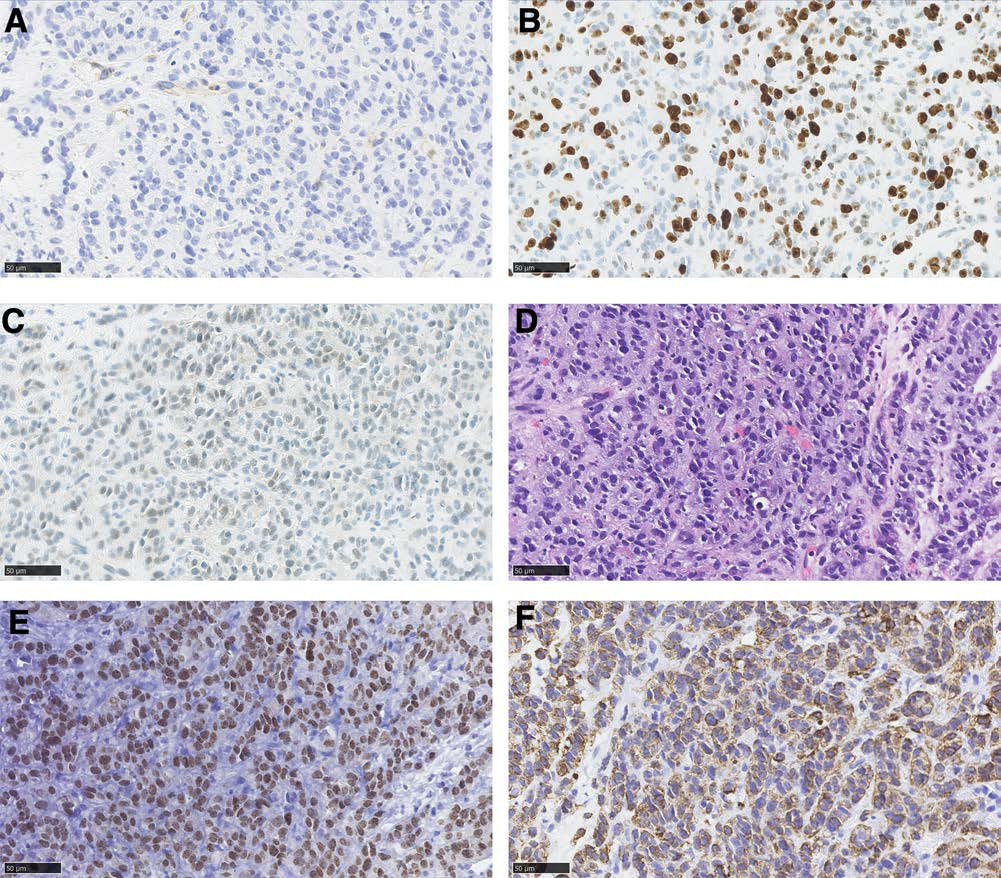
Immunohistochemical results on TdT, Ki-67, S-100, H&E, NUT, CK5/6. (A) TdT (10*40). (B) Ki-67 (10*40). (C) S-100 (10*40). (D) H&E (10*40). (E) NUT (10*40). (F) CK5/6 (10*40).

**Figure 4.**
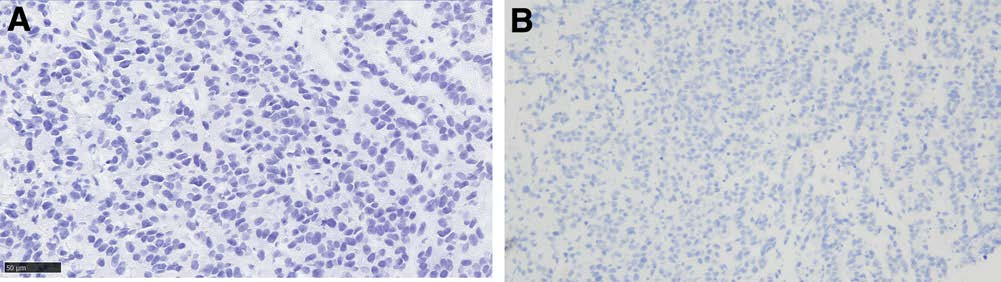
Immunohistochemical results on Syn, PD-L1. (A) Syn (10*40). (B) PD-L1 (10*20).

In November 2021, a 27-year-old female presented to the hospital with symptoms of intermittent coughing, chest tightness, and abdominal distension persisting for more than a month. A computed tomography (CT) scan performed on November 28 revealed metastases in the right hilar and mediastinal lymph nodes, a malignant mass in the right hilar region and the lower middle lobe of the right lung (Fig. 1), pelvic and abdominal effusion, and bilateral cystic, solid tumors in the adnexal region that may be cystic adenocarcinomas (Fig. 2). Tumor marker analysis showed elevated levels of alpha-fetoprotein (56.8 ng/ml; reference range, 0–25 ng/mL) and cancer antigen-125 (87.2 U/mL; reference range, 0–35 U/mL). Imaging consultation on the same day identified that the lesion was associated with enlarged lymph nodes in the right hilum and mediastinum, and the nondistended tissue of the right lower lobe of the lung. The overall maximum cross-sectional area size was 10.0*5.5 cm, and it encompassed the right lower pulmonary vein. Furthermore, multiple irregular cystic, solid masses with a maximum cutoff area of approximately 20.0*13.0 cm were detected in the abdominal and pelvic cavities in the bilateral adnexal regions, suspected to be metastases. Pathological examination revealed a poorly differentiated carcinoma with features including necrosis, neutrophil infiltration, focal keratinization, and immunohistochemical findings consistent with NC (Figs. 3 and 4). Both the clinical and immunohistochemical findings suggest that the cancer has metastasized from the lungs to the pelvic area. Immunohistochemistry is given as follows: cytokeratin (AE1/AE3) (+), DeltaNp63 (P40) (+), cytokeratin 7 (−), paired box 8 (−), thyroid transcription factor-1 (−), marker of proliferation Ki-67 (+, 60%), smooth muscle actin-auto (+, partial), cluster of differentiation 34 (−), spalt-like transcription factor 4 (−), cyclin-dependent kinase inhibitor 2A (+, little), desmin (−), integrase interactor 1 (+), smooth muscle myosin monoclonal antibody (−), S100 protein-auto (−), cytokeratin antibody 5.2 (−), NUT (+), terminal-deoxynucleotidyl transferase (+), synaptophysin (−), and cytokeratin 5/6 (+). Further testing using fluorescence in situ hybridization revealed positive results for NUT. Based on all of the above information, a diagnosis of stage IV lung cancer (NC) with metastases in the hilar and mediastinal lymph nodes and pelvic region was made.

**Figure 5.**
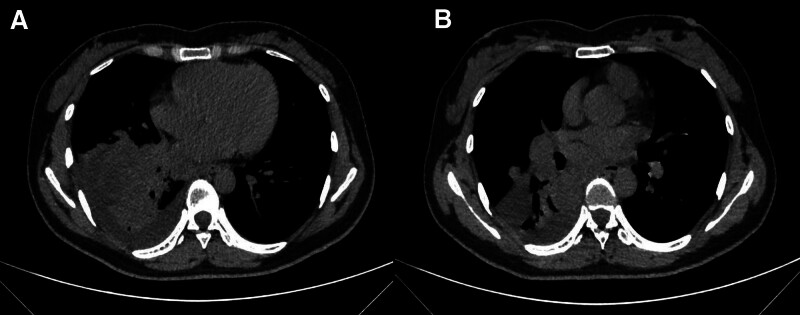
January 5, 2022. Lungs computed tomography: malignant tumor of the right lung fuller than before. Right hilar and mediastinal lymph node metastasis, fuller than before; right pleural effusion. (A) Maximum cross-section of tumor. (B) Cross-section similar to Figure 1B.

**Figure 6.**
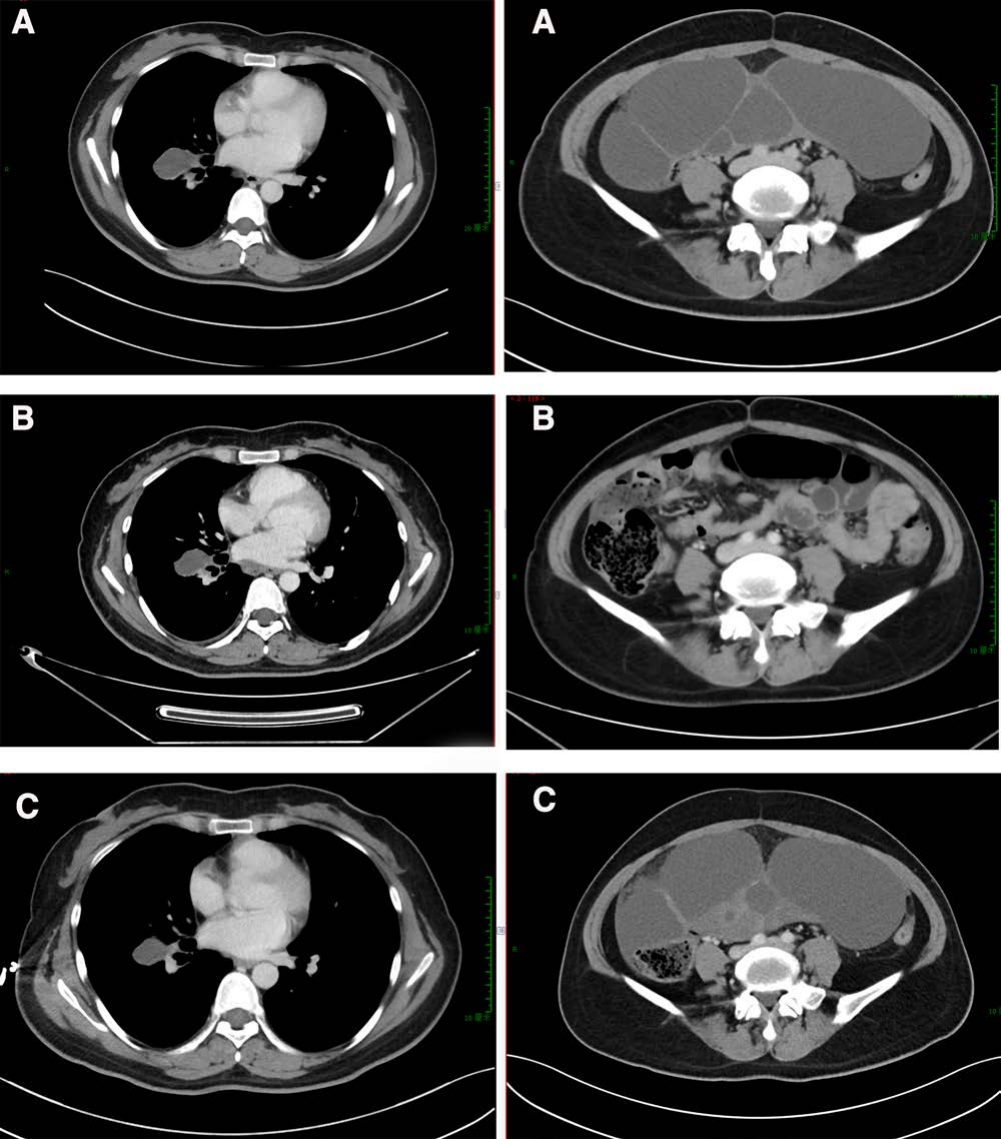
Computed tomography: February 25, 2022; May 23, 2022; and July 4, 2022. (A) May 25, 2022, cross-section of lung and pelvic tumors. (B) May 23, 2022, cross-section of lung and pelvic tumors. (C) July 6, 2022, cross-section of lung and pelvic tumors.

On December 15, 2021, the patient received cycle 1 chemotherapy, which included the following regimen: tislelizumab 200 mg d1 + paclitaxel liposome 240 mg d1 + nedaplatin 60 mg d2 to d 3. A subsequent chest CT on January 5, 2022, revealed an increase in the size of the right lung malignancy, as well as enlargement of the right hilar and mediastinal lymph node metastases and an increase in right pleural effusion (Fig. 5). Tumor markers were elevated, with cancer antigen-125 at 433.00 (0–35) U/mL, cytokeratin fragment 21-1 at 6.13 (0–3.3) ng/mL, and carbohydrate antigen 242 at 20.29 (0–20) U/mL, indicating progressive disease. Patients subsequently received 4 cycles of second-line therapy, albumin paclitaxel 450 mg d1 + cisplatin 40 mg d1 to d 3 + bevacizumab 500 mg d1:quaque 21 die = once every 21 days. Imaging evaluations on April 23 and May 23, 2022, showed a reduction in the size of the right lung mass, hilar and mediastinal lymph nodes, and bilateral adnexal pelvic masses (Fig. 6), indicating stable disease. Cycles 5, 6, and 7 were administered on May 6, May 27, and June 17, 2022. According to the CT from July 2022 (Fig. 6), the mass in the patient’s right lung had not changed compared with the previous scan. However, the lymph nodes in the mediastinal and hilar regions, as well as the pelvic masses in the bilateral adnexal areas, had increased in size. Unfortunately, the patient developed intestinal obstruction in October 2022 and was treated conservatively. The patient passed away on November 7, 2022.

## 3. Discussion

NUT midline carcinoma is an infrequent and notably aggressive malignancy.^[6]^ Initially thought to predominantly affect adolescents, subsequent research has established that it can manifest across all age groups and is not restricted by gender.^[7]^ However, due to the lack of cohort studies or large sample sizes, the pathophysiology of NC remains unknown. Recent investigations have indicated that while a history of smoking is common among individuals with primary NC of the lung, nonsmokers may have a heightened susceptibility to this disease.^[8]^ We have collated the characteristics of the relevant case reports and summarized them in Table 1.

**Table 1:** Characteristics and treatment of the lung nuclear protein in testis carcinoma.

No.	Reference	Sex	Age	Smoking, yr	Treatment	OS, mo
1	[9]	Male	25	7	EP→TIP	N/A
2	[10]	Male	57	No	DP→nivolumab	4
3	[11]	Male	36	27	VIP→radiotherapy	10
4	[12]	Male	19	Yes	EP + radiotherapy	18
5	[13]	Male	34	7.5	Carboplatin radiotherapy pembrolizumab	N/A
		Male	44	10	Surgery + TP	N/A
		Female	48	No	Carboplatin Pembrolizumab Surgery Pembrolizumab	N/A
		Male	43	20	Surgery + TP	N/A
		Female	18	No	TP	N/A
6	[14]	Female	63	N/A	Chemotherapy	13*
7	[15]	Male	30	No	Chemotherapy	12*
8	[16]	Female	31	N/A	Surgery	1
9	[17]	Male	40	No	EP→SSG IV→VAI→radiotherapy→anlotinib→Bevacizumab→paclitaxel→PD-1 inhibitor corrida (drug K)	4.7
10	[18]	Male	49	No	TP + radiotherapy + durvalumab→molibresib→gamma knife radiosurgery→carboplatin-pemetrexed-pembrolizumab	16
11	[19]	Male	70	No	EP + radiotherapy + bevacizumab→sindilizumab→anlotinib	24*
12	[4]	Male	14	No	IRS III→ICE→surgery→VP-16 + L-PAM	12
		Female	7	No	TP + radiotherapy + PAC	4
13	[20]	Female	36	No	DP + radiotherapy	9*
14	[21]	Male	16	N/A	Surgery + chemotherapy	3
15	[22]	Male	27	No	TP + radiotherapy	5
16	[23]	N/A	27	No	Chemotherapy	8
17	[24]	Male	28	Yes	TP + pembrolizumab + surgery	12
18	[25]	Male	60	48	Surgery	6
19	[26]	Male	69	N/A	Surgery + EP + bevacizumab	10*
20	[27]	Male	36	No	VIP→Radiotherapy + GSK525762	N/A
21	[28]	Male	33	18	Anlotinib + radiotherapy	4

DP = docetaxel-cisplatin, EP = etoposide-cisplatin, ICE = ifosfamide + carboplatin + etoposide, VP-16 = etoposide, IRS Ⅲ = vincristine+ etoposide + cyclophosphamide + cisplatin, L-PAM = L-Phenylalanine mustard, N/A = not available, OS = overall survival, SSG IV = VAI (vincristine + epirubicin + ifosfone amide) and PAI (cisplatin + epirubicin + lfosfamide), TIP = paclitaxel, cisplatin and ifosfamide, VIP = cisplatin, ifosfamide, and etoposide.

* Up to the last follow-up visit.

Patients with primary pulmonary NC often present with symptoms similar to lung cancer and may even be asymptomatic.^[13]^ Common symptoms include cough, dyspnea, chest pain, and hemoptysis during the initial visit.^[29]^

Diagnosing NC requires a combination of imaging and pathology, as neither method alone provides enough specificity to make a conclusive diagnosis.^[30]^ Therefore, a combination of the 2 is required. Radiographically, NC mirrors squamous cell carcinoma in its propensity for aggressive invasion and encroachment on adjacent tissues,^[31]^ with a potential for necrosis and metastasis.^[32]^ Despite squamous cell carcinoma being rare in adolescents, the presence of such histological features in tumors of this demographic warrants consideration of NC.^[33]^ An examination of 55 patient reports highlighted that NC typically presents as substantial, irregularly shaped soft tissue masses with occasional pleural involvement and potential lymphatic or distant metastases.^[29]^ Statistically, these tumors predominantly occupy the left or lower right pulmonary lobes, often associated with pleural effusion, compressive atelectasis, or lung necrosis. On magnetic resonance imaging, NC usually shows a low signal on T1-weighted imaging and a high signal on T2-weighted imaging. Nonenhancing necrotic areas within the tumor can also be identified, and enhancement is heterogeneous.^[34]^ Given the highly aggressive nature of NC and its propensity for distant metastases, comprehensive positron emission tomography/CT scanning is imperative for the identification of additional infiltration sites, tumor staging, and early detection of metastases.^[35]^ While imaging can offer diagnostic hints, definitive diagnosis hinges on pathological evaluation. NC is histologically akin to poorly differentiated squamous cell carcinoma, with distinctive pathological features including variable cell sizes, focal abrupt keratinization^[36,37]^ (noted in ≈30% of cases),^[5]^ scant hyaline cytoplasm, and large, variably shaped nuclei with prominent nucleoli and granular to coarse chromatin.^[37,38]^ NC is frequently misdiagnosed as Ewing sarcoma, leukemia, or other diseases because it lacks distinctive pathological symptoms.^[6,39]^ Neutrophil infiltration is another pathogenic feature of NC.^[40]^ Most primary pulmonary NC cases were positive for cytokeratins (AE1/AE3, cytokeratin antibody 5.2, cytokeratin 5/6, and pan-cytokeratin were more common), P63, P40, and NUT.^[16,41,42]^ Thyroid transcription factor-1 and a variety of neuroendocrine markers were mostly negative. Marker of proliferation Ki-67, epithelial membrane antigen, and MYC proto-oncogene were positive in some cases.^[38]^ This is broadly consistent with the results for this patient. Recent advancements in diagnostic techniques, such as immunohistochemistry using the anti-NUT monoclonal antibody C52,^[43]^ fluorescence in situ hybridization, reverse transcription-polymerase chain reaction, and gene sequencing, alongside conventional cytogenetic karyotyping, have enhanced the diagnostic accuracy for NC, facilitating more targeted therapeutic interventions.^[38]^

Currently, there is no consensus on a standard treatment protocol for NC. Doctors typically employ a combination of surgery, chemotherapy, and radiotherapy to manage the disease,^[1]^ with some experts also exploring targeted therapy and immunotherapy.^[44,45]^ Early radical surgery improves progression-free survival (PFS) and OS in patients with NC.^[38,45,46]^ A study by Chau et al^[46]^ involving 48 patients, half of whom underwent surgical intervention, highlighted a significant improvement in 2-year OS rates for the surgical group compared with their nonsurgical counterparts. For those ineligible for surgical treatment, radiotherapy emerges as a viable alternative, albeit with considerations for the rapid progression characteristic of the disease post-treatment.^[38]^ The chemotherapeutic regimen typically includes agents such as platinum, paclitaxel, cyclophosphamide, and etoposide, with survival outcomes varying significantly among patients.^[4,47]^ Moreover, radiotherapy has demonstrated potential in prognostic enhancement,^[5,45]^ with higher radiation doses correlating with improved treatment results.^[48]^

With the widespread use of immunotherapy in the treatment of NC, an increasing number of patients are beginning to use immunotherapy as a second-line or follow-up treatment to improve the disease’s prognosis.^[1,49]^ Several cases of longer survival time with immunotherapy were reported in a study by Li et al.^[38]^ Reports of extended survival times with immunotherapeutic agents such as navollizumab^[38,50]^ and pembrolizumab^[1,13,44,51]^ underscore the potential of immunotherapy in improving patient outcomes, particularly when integrated with conventional treatments. Based on the above literature reports and the long trailing effect of immunotherapy, the patient’s first-line treatment was chosen to add tislelizumab to conventional chemotherapy; however, the patient’s disease progressed rapidly after 1 cycle and did not achieve the expected effect. To determine why this was happening, the patient was tested for immune-related indicators such as programmed cell death-ligand 1 expression levels, and the results showed that the tumor cell positive percentage fraction was 1% and was negative (Fig. 5). Primary resistance is characterized by a lack of antigenic mutations, loss of tumor antigen expression, loss of human leukocyte antigen expression, altered antigen processing mechanisms, and altered expression of several signaling pathways (mitogen-activated protein kinase, phosphoinositide 3-kinase, wingless-related integration site, and interferon), as well as constitutive programmed cell death-ligand 1.^[52]^ Other suppressive immune cells in the tumor microenvironment, such as regulatory T cells, myeloid suppressor cells, and M2 tumor-associated macrophages, are extrinsic tumor resistance factors.^[53]^

In the realm of targeted therapy, bevacizumab has emerged as a noteworthy agent, leveraging its antiangiogenic properties to inhibit tumor growth and neovascularization by targeting the vascular endothelial growth factor pathway. This therapeutic strategy, alongside other antiangiogenic drugs, has shown efficacy in controlling tumor progression, offering a promising treatment alternative for NC.^[26]^ Furthermore, 2 patients with orbital NC who had undergone surgery received third-line anlotinib treatment and lived for more than 15 and 8 months, respectively.^[54,55]^ Following the progression of first-line immunotherapy, the patients were given chemotherapy in combination with bevacizumab. The patient, thus, had a PFS of up to 6 months, significantly longer than the 6.5-month median survival for pulmonary NC.

In addition, the exploration of new targeted agents, particularly bromodomain and extra-terminal domain (BET) inhibitors, in clinical trials has revealed preliminary efficacy, with some patients achieving partial remission or stable disease states.^[56]^ According to the findings of 2 phase I/II clinical trials of the BET inhibitor molibresib (GSK525762 and NCT01587703), 4 patients with NC achieved partial remission, 8 were considered stable, and 4 had PFS time of >6 months. Another clinical trial of BET inhibitors (OTX015/ MK-8628) enrolled 4 patients, and 2 had rapid tumor regression with the drug and OS significantly longer than 6.7 months (19 and 18 months, respectively). There are also some BET inhibitor drugs in clinical trials, such as BAY1238097, GSK2820151, TEN-010, and BI 894999, and the results of the relevant clinical studies can be followed up on.^[44,57–59]^

Despite these therapeutic advancements, NC remains a highly aggressive disease with a dismal prognosis, characterized by a median survival time of merely 6.7 months.^[5]^ Saiki et al^[60]^ found that the average survival time after diagnosis was 6.75 (±standard deviation, 4.60) months, which closely aligns with the International NUT registry (6.5 months). The disease is frequently diagnosed at advanced stages,^[61]^ complicating treatment efforts and underscoring the urgent need for effective therapeutic strategies and early detection methods.

## 4. Conclusion

The individual persevered for nearly a year following their diagnosis before their eventual passing on November 7, 2022, surpassing the typical survival time for individuals with pulmonary NC. Moreover, they experienced 6 months of PFS, thanks to second-line therapy. This instance illustrates the promising application of antivascular endothelial growth factor analogs as a potential treatment approach in the times ahead.

## Author contributions

**Writing – original draft:** Ran Li

**Investigation:** Ye Zhang

**Resources:** Qian Liu

**Writing – review & editing:** Aiqin Gao, Qi Dang
